# Approximately Half of Total Protein Intake by Adults Must be Animal-Based to Meet Nonprotein, Nutrient-Based Recommendations, With Variations Due to Age and Sex

**DOI:** 10.1093/jn/nxac150

**Published:** 2022-07-11

**Authors:** Florent Vieux, Didier Rémond, Jean-Louis Peyraud, Nicole Darmon

**Affiliations:** MS-Nutrition, Marseille, France; Université Clermont Auvergne, INRAE UNH, Clermont-Ferrand, France; UMR INRAE-ACO Pegase, St Gilles, France; MoISA, University of Montpellier, CIRAD, CIHEAM-IAMM, INRAE, Institut Agro, IRD, Montpellier, France

**Keywords:** animal-to-plant protein ratio, nutritional adequacy, optimization, protein quality, affordability, diet cost, France

## Abstract

**Background:**

Shifting towards a more plant-based diet, as promoted in Western countries, will reduce the animal protein contribution to total proteins. Such a reduction may not only impair protein adequacy, but also the adequacy in other nutrients.

**Objectives:**

We determined, for different adult subpopulations, the minimum total protein levels and the minimum animal protein contributions to total proteins that are compatible with the fulfillment of all nonprotein nutrient-based recommendations.

**Methods:**

Mean nutritional contents and mean diet costs were estimated using a French, cross-sectional, representative survey for 5 French subpopulations: *1*) women < 50 y; *2*) women 50–64 y; *3*) women ≥ 65 y; *4*) men < 65 y; and *5*) men ≥ 65 y. For each subpopulation, linear programming optimization was used to assess the minimum protein level (model set #1) and the minimum animal protein contribution to total proteins (model set #2) that are compatible with the fulfillment of all nutrient-based recommendations (except proteins, for which levels were analyzed as outputs). Total diet costs were not allowed to increase. Eating habits were considered in model set #2 only.

**Results:**

The minimum amount of protein that was theoretically compatible with the fulfillment of nutrient-based recommendations (model set #1) was below the minimum recommended protein intake for all subpopulations except women < 50 y. In model set #2, for women and men ≥ 65 y, decreasing animal protein contributions to total proteins below 55% and 60%, respectively, led to protein levels below recommended levels. For the other subpopulations (women < 50 y, women 50–64 y, and men < 65 y), the lowest animal protein contributions to total proteins compatible with a nutritionally adequate diet (including protein adequacy) were 55%, 50%, and 45%, respectively.

**Conclusions:**

This study provides factual information about the animal protein contributions to total proteins compatible with meeting all nutrient-based recommendations at no additional cost, and shows that they vary between 45% and 60% depending on the group of adults considered.

## Introduction

Guiding principles for sustainable and healthy diets include eating an abundance and a variety of minimally processed, plant-based foods (fruits, vegetables, nuts, unrefined cereals, legumes, etc.) and moderate amounts of animal-based foods (1). Higher consumption of plant-based foods has consistently been associated with positive health outcomes (2), whereas excess consumption of red meat and processed meat is often discouraged by public health nutrition guidelines (3).

Because meat has a higher protein content per kilocalorie than plant-based foods, shifting towards more plant-based diets and less meat will inevitably reduce both total dietary proteins and the animal protein contributions to total proteins (4). Animal proteins have a more balanced indispensable amino acid profile and greater digestibility than plant proteins, although plant proteins are commonly depicted as being of lower quality than animal proteins (5). Yet, in Western countries, where the quantities of protein consumed generally exceed minimum requirements, quality differences between proteins from different food sources have negligible impacts on protein and amino acids adequacies (4, 6). In other words, for the majority of people in Western countries, who are typically omnivorous, the risk of inadequate protein intake is low, and it is minimally influenced by the animal protein contribution to the diet. Yet, caution is warranted for elderly people because they have higher protein requirements than other adults (estimated at 1 g/kg for adults over 65 y and 0.83 g/kg for younger adults) (7). Thus, for French adults aged 65 and over, a higher risk of frailty was observed for protein intakes lower than 1 g/kg of body weight, independent of energy intake levels (8).

Protein-source foods provide several nutrients other than proteins and largely contribute to overall nutrient adequacy (9, 10). Animal-based foods provide nutrients that are either not found in plant-based sources (such as vitamins D and B12 and long-chain omega 3 fatty acids), found in small amounts (e.g., vitamin B6 and riboflavin), or found in less bioavailable forms (e.g., iron and zinc), with the latter including unique sources of fiber, folate, vitamins E and C, and other antioxidants. In addition, diets with plenty of plant-based foods are not necessarily more affordable than diets with more animal-based foods (11). More generally, food budget constraints are important determinants of food choices, and healthy food choices tend to be more expensive than unhealthy ones (12).

The goal of this study was to determine the extents to which decreases in protein intakes, particularly from animal-based sources, could be made without impairing the nutritional adequacy of the diet and at no additional cost. To reach this goal, mathematical optimization models were developed to determine the theoretical minimum levels of total dietary proteins, as well as the minimum percentages of animal proteins in total proteins that are compatible with the fulfillment of all nutrient-based recommendations, without changing the total energy content and at no additional cost, for 5 subpopulations of French adults differing according to sex and age.

## Material

### Populations of interest

Five subpopulations of adults were defined according to nutrient recommendation levels. First, segmentation was applied based on sex because recommended levels of vitamins (A, E, B6), minerals (magnesium, zinc), and water differ for men and women (**Table 1**). Then, for each sex, an age threshold of 65 y was used in order to take into account the differences in recommended levels of protein intake: 1.0 g of proteins/kg of body weight for adults aged 65 years old and older and 0.83 g of proteins/kg of body weight for younger adults (7). The subpopulation of women was further divided according to iron needs, considering 50 y as the menopausal year (13). This led to 5 subpopulations, as follows: *1*) women < 50 years old; *2*) women 50–64 years old; *3*) women ≥ 65 years old; *4*) men < 65 years old; and *5*) men ≥ 65 years old.

**TABLE 1:** Constraints applied to model set #1 and model set #2, for each subpopulation^1^

	Women			Men	
Subpopulation	<50 y	50–64 y	≥65 y	<65 y	≥65 y
Nutritional constraints (applied to model set #1 and model set #2)					
Energy, kcal/d	= Observed (1731.6)	= Observed (1711.5)	= Observed (1675.4)	= Observed (2207.1)	= Observed (2082.8)
Carbohydrates, % energy	[40; 55]	[40; 55]	[40; 55]	[40; 55]	[40; 55]
Fats, % energy	[35; 40]	[35; 40]	[35; 40]	[35; 40]	[35; 40]
Linoleic acids, % energy	≥4	≥4	≥4	≥4	≥4
Alpha-linolenic acids, % energy	≥1	≥1	≥1	≥1	≥1
DHA + EPA, mg/d	≥250	≥250	≥250	≥250	≥250
SFA, % of energy	≤12	≤12	≤12	≤12	≤12
Lauric + myristic + palmitic acids, % energy	≤8	≤8	≤8	≤8	≤8
Total sugars, without lactose, mg/d	≤100	≤100	≤100	≤100	≤100
Sodium, mg/d	≤ Observed (2613.0)	≤ Observed (2624.7)	≤ Observed (2689.1)	≤ Observed (3459.9)	≤ Observed (3575.5)
Sodium-to-potassium ratio, molar	≤1	≤1	≤1	≤1	≤1
Water, ml/d	2000	2000	2000	2500	2500
Fibers, g/d	≥30	≥30	≥30	≥30	≥30
Vitamin A, μg/d	[650; 3000]	[650; 3000]	[650; 3000]	[750; 3000]	[750; 3000]
Thiamin, mg/MJ	≥0.14	≥0.10	≥0.10	≥0.14	≥0.10
Vitamin B12, μg/d	≥4	≥4	≥4	≥4	≥4
Riboflavin, mg/MJ	≥0.17	≥0.17	≥0.17	≥0.17	≥0.17
Niacin, mg NE/MJ;mg/d	[1.6; 900]	[1.6; 900]	[1.6; 900]	[1.6; 900]	[1.6; 900]
Pantothenic acid, mg/d	≥4.7	≥4.7	≥4.7	≥5.8	≥5.8
Vitamin B6, mg/d	[1.5; 25]	[1.6; 25]	[1.6; 25]	[1.8; 25]	[1.7; 25]
Folates, μg/d	≥330	≥330	≥330	≥330	≥330
Vitamin C, mg/d	≥110	≥110	≥110	≥110	≥110
Vitamin D, μg/d	[5; 50]	[5; 50]	[5; 50]	[5; 50]	[5; 50]
Vitamin E, mg/d	[9.9; 300]	[9.9; 300]	[9.9; 300]	[10.5; 300]	[10.5; 300]
Calcium, mg/d	[960; 2500]	[950; 2500]	[950; 2500]	[960; 2500]	[950; 2500]
Copper, mg/d	[1.0; 5.0]	[1.3; 5.0]	[1.3; 5.0]	[1.3; 5.0]	[1.6; 5.0]
Iron, mg/d	≥16^2^	≥11	≥11	≥11	≥11
Iodine, μg/d	[150; 600]	[150; 600]	[150; 600]	[150; 600]	[150; 600]
Magnesium, mg/d	≥360	≥360	≥360	≥420	≥420
Phosphorus, mg/d	≥700	≥550	≥550	≥700	≥550
Selenium, μg/d	[70; 300]	[70; 300]	[70; 300]	[70; 300]	[70; 300]
Zinc, mg/d	[7.5; 25]	[7.5; 25]	[7.5; 25]	[9.4; 25]	[9.4; 25]
	≥0.0058 × phytates + 5.8	≥0.0058 × phytates + 5.8	≥0.0058 × phytates + 5.8	≥0.0077 × phytates + 7.1	≥0.0077 × phytates + 7.1
Nonnutritional constraints (applied to model set #1 and model set #2)					
Diet cost, €/d	≤ Observed (5.16)	≤ Observed (5.77)	≤ Observed (5.52)	≤ Observed (6.33)	≤ Observed (6.30)
Fish, g/d	<28.6	<28.6	<28.6	<28.6	<28.6
Quantity per food item,^3^ g/d	≤ 95th pctl	≤ 95th pctl	≤ 95th pctl	≤ 95th pctl	≤ 95th pctl
Fortified products and mineral waters,^4^ g/d	≤ Observed	≤ Observed	≤ Observed	≤ Observed	≤ Observed
Additional constraints applied to model set #2 only					
Quantity per food group,^5^ g/d	[5th pctl; 95th pctl]	[5th pctl; 95th pctl]	[5th pctl; 95th pctl]	[5th pctl; 95th pctl]	[5th pctl; 95th pctl]
Quantity per food subgroup,^5^ g/d	[5th pctl; 95th pctl]	[5th pctl; 95th pctl]	[5th pctl; 95th pctl]	[5th pctl; 95th pctl]	[5th pctl; 95th pctl]
Quantity per food category,^5^ g/d	[5th pctl; 95th pctl]	[5th pctl; 95th pctl]	[5th pctl; 95th pctl]	[5th pctl; 95th pctl]	[5th pctl; 95th pctl]
Animal protein contributions to total proteins, %	Gradual decrease by 5% steps	Gradual decrease by 5% steps	Gradual decrease by 5% steps	Gradual decrease by 5% steps	Gradual decrease by 5% steps

1 Model set #1 focused on determining the theoretical minimum level of total dietary proteins compatible with the fulfillment of all nutrient-based recommendations (without imposing a minimum quantity of total proteins), without changing the total energy content and at no additional cost. Model set #2 focused on determining the minimum percentage of animal proteins in total proteins compatible with the fulfillment of all nutrient-based recommendations (without imposing a minimum quantity of total proteins), without changing the total energy content, at no additional cost, and taking eating habits into account. Values in brackets are ranges e.g., in the case of fats, the recommended amount is between 35 and 40% of energy. Abbreviation: pctl, percentile.

2 Under the assumption of high iron losses through menstrual blood.

3 Quantities < 95th percentile estimated only for consumers of the food item.

4 Fortified foods were defined as breakfast cereals (3 food items), multivitamin juice, soya-based drink, tomato soup, soya-based desserts (3 food items), cocoa powder, pineapple juice, a mixture of oils, instant drink, and low-fat margarine. Each food item was constrained to remain below its observed amount. Mineral waters (8 food items) were constrained in the same way to favor tap water.

5 The 5th percentile and 95th percentile were estimated for all individuals, including nonconsumers of the food group.

### Dietary data and calculation of mean observed diets

Dietary data were derived from the Second French Individual and National Study on Food Consumption (INCA2), a 7-day open-ended food-record representative survey conducted between 2005 and 2007 by the French Agency for Food, Environmental, and Occupational Health and Safety (14). For this study, all adults above the age of 18 y were studied, leading to a total sample of 2624 individuals, distributed as 922 women < 50 y, 418 women between 50 and 64 y, 197 women ≥ 65 y, 936 men < 65 y, and 151 men ≥ 65 y. The INCA2 survey was approved by the French National Commission for Computed Data and Individual Freedom (Commission Nationale de l'Informatique et des Libertés, CNIL).

For each of the 5 subpopulations, we estimated the average daily dietary intake, also called the observed diet below, based on 212 frequently consumed food items (**Supplemental Table 1**), following a previously described matching methodology (15). Based on the French food composition table [CIQUAL 2013 (16)], a sex-specific nutrient composition database containing energy, fiber, fats (including linoleic and alpha-linolenic acids; EPA; DHA; and lauric, myristic, and palmitic acids, as well as total SFA), proteins (including amino acids), carbohydrates, 11 vitamins, and 11 minerals was derived for each of the 212 food items (15). Phytate and amino acid contents in foods were derived from the International Network of Food Data Systems databases (15). Average prices for the 212 food items were calculated based on data from the 2006 Kantar Worldpanel (15, 17). After removal of alcoholic items from food consumption, composition, and price databases, nutrient intakes and costs associated with the consumption of 207 food items were estimated for each subpopulation, leading to 5 observed diets.

### Modeling

The models developed in this study used a mathematical optimization technique based on the simplex algorithm. An optimization model is comprised of variables, constraints, and an objective function, and the algorithm finds the value that each variable must have to comply with the constraints while optimizing (i.e., minimizing or maximizing) the value given by the objective function. In diet optimization models, the variables are the quantities of foods, and the algorithm finds the quantity of each food in the modeled diet that is compatible with the simultaneous fulfillment of all constraints for the minimum (or maximum) value of the objective function.

In the present study, 2 kinds of models were developed, called model set *#*1 and model set #2. Model set #1 diets were developed to determine the minimum protein contents compatible with the fulfillment of all nutrient recommendations (without imposing a minimum amount of total proteins), without changing the observed energy content and without exceeding the observed diet cost. Model set #2 diets were aimed at determining the minimum percentages of animal proteins in total proteins compatible with the fulfillment of all nutrient-based recommendations without changing the observed energy content, without exceeding the diet cost, and taking eating habits into account.

#### Variables

Variables were the same for the 2 kinds of models. All 207 food items in the subpopulation-specific database were the variables for each model performed.

#### Constraints

Constraints applied to models are described in Table 1. Nutritional constraints were applied to both model set #1 and model set #2. Each model included a subpopulation-specific set of nutritional constraints imposing the fulfillment of all nutrient-based recommendations (except proteins, for which levels were analyzed as output). The energy contents of modeled diets were kept equal to those of observed diets (i.e., modeled diets were isoenergetic with observed diets). All nutrient reference values (NRV) were taken from the French Agency for Food, Environmental and Occupational Health and Safety reports (7, 13) available at the start of the project. EPA + DHA and vitamin D constraints were adapted because they were too constraining (official recommendations are 500 mg/d for EPA + DHA and 15 μg/d for vitamin D). The EPA + DHA intake was set at 250 mg/d, in agreement with the European Food Safety Authority dietary reference value (18), and the vitamin D constraint was set at 5 μg/d, because this intake is considered as adequate by various official authorities (19). As the recommendations for calcium differ before and after the age of 25 y, NRV were weighted according to the percentages of individuals older and younger than 25 y among women under the age of 50 and men under the age of 65. Sodium (no quantified recommendation in France) was required to be maintained below observed intake levels. In accordance with the French NRV, the recommended levels of zinc applied to the modeled diets depended on the levels of phytates in diets. In accordance with French food-based dietary guidelines, the content of fish was maintained at less than 200 g/wk to limit fish-related contaminant exposure.

In both model set #1 and model set #2, an applied constraint where the diet cost was kept below or equal to the observed diet cost was applied to all models, in order to consider the realism and affordability of the modeled diets. A maximum quantity was applied to all models for each of the 207 food items, to avoid including an unrealistic amount for a given food item.

Other constraints were only applied to model set #2. Consumption constraints, applied as minimum and maximum amounts by food group, subgroup, and categories, were introduced in order to better accommodate current eating habits in diets obtained with model set #2. Moreover, model set #2 diets were used to find the minimum percentages of animal proteins in total proteins that are compatible with the fulfillment of nutrient-based recommendations. To do so, a constraint was introduced and progressively strengthened to reduce, by 5% steps, the percentage of animal proteins in total proteins, leading to a group of different model set #2 diets for each subpopulation. The first model of the set did not include any constraint on animal protein contributions to total proteins. Then, starting from the percentage of animal proteins in total proteins reached in the first model, that percentage was progressively reduced by 5% steps to obtain the other model set #2 diets until no solution could be found.

#### Objective function

In model set #1, total proteins (in grams) were minimized by applying the following objective function:

(1) \begin{eqnarray*} Min\,Prot = \sum\limits_{i = 1}^{207} {Q_i^{opt}*Pro{t}_i} \end{eqnarray*}

Here, *Prot* is the protein quantity in the modeled diet, Q^opt^_i_ is the modeled quantity of food *i*, and *Prot_i_* is the protein content (in grams) of food *i*.

In model set #2, the objective function was designed to minimize the deviation from the observed diet in order to maximize acceptance, and was calculated as the sum of absolute differences between the quantity of each food from the 207 foods listed in the observed diet and the quantity of each food selected in the modeled diet, as follows:

(2) \begin{eqnarray*} Min\,D = \sum\limits_{i - 1}^{207} {\left| {Q_i^{opt} - Q_i^{obs}} \right|} \end{eqnarray*}

Here, *D* is the absolute departure (in grams) between the modeled and observed diets, Q^opt^_i_ is the modeled quantity of food *i*, and Q^obs^_i_ is the observed quantity of food *i* (estimated over the whole subpopulation sample, including nonconsumers).

### Selection of 1 model set #2 diet for each subpopulation

For each subpopulation, among the model set #2 diets, the diet with the lowest animal protein contribution to total proteins, compatible with nutrient and protein adequacy, affordability, and eating habits, was selected by keeping the diet that followed the 3 following nutritional and consumption criteria:

1. Adequate total protein content: the total protein content must not fall below the minimum level recommended for that subpopulation (so that the selected diet is fully nutritionally adequate because it fulfills all nutrient-based recommendations, including the recommendation for proteins);
2. Limited food mass deviation from the observed diet: the objective function value (i.e., the sum of absolute differences between the modeled and observed quantities of each food) must be lower than the total mass of the observed diet (in kilograms); and
3. Limited exclusion of food items in the modeled diet: the total number of foods does not drop by 15% or more compared to the previously modeled diet (i.e., the diet modeled with a 5-point higher percentage of animal proteins in total proteins).

### Protein adequacy

It was considered that the diets (observed and modeled) displayed protein adequacy when: *1*) the total protein content was higher than the recommended amount for the subpopulation; *2*) the quality of indispensable amino acids (histidine, isoleucine, leucine, lysine, methionine + cysteine, phenylalanine + tyrosine, threonine, tryptophan, valine) was adequate; and *3*) the quantity of indispensable amino acids was adequate.

Qualitative and quantitative assessments of amino acid contents were performed by comparing their contents with recommended amounts (**Supplemental Table 2**), expressed in 2 ways. First, we assessed the respective recommended amounts by considering the digestible protein content (quality):

(3) \begin{eqnarray*} {\rm{\ Quali\ A}}{{\rm{A}}}_i = \frac{{A{A}_i}}{{AArec{o}_i \times Prot\ DIG}}\ \times 100 \end{eqnarray*}

Here, *Quali AA_i_* is the quality indicator of indispensable amino acid *i* in the diet, *AA_i_* is the diet's digestible amino acid content *i* (mg/d), *AAreco_i_* is the recommended amount of indispensable amino acid *i* (mg/g of proteins), and *Prot DIG* is the digestible protein content in the diet (g/d).

Second, we assessed the respective recommended amounts in the context of a diet achieving the minimum recommended amount of total proteins (quantity):

(4) \begin{eqnarray*} {\rm{Quantity}}\,\,{\rm{A}}{{\rm{A}}}_{\rm{i}} = \frac{{A{A}_i}}{{AArec{o}_i \times Prot\ reco}}\ \times 100 \end{eqnarray*}

Here, *Quantity AA_i_* is the indicator of indispensable amino acid quantity *I* in the diet, *AA_i_* is the diet's digestible amino acid content *i* (mg/d), *AAreco_i_* is the recommended amount of indispensable amino acid *i* (mg/g of proteins), and *Prot reco* is the recommended amount of total proteins (expressed in g/d), with the latter being a function of the average body weight of the subpopulation.

### Analyses

Analyses were repeated for each of the 5 subpopulations. First, the minimum total protein content achievable with model set #1 (i.e., the lowest theoretical protein level compatible with the fulfillment of all nutrient-based recommendations, except the recommendation on proteins, for which levels were analyzed as output, at no additional cost) was reported and compared to both the recommended and observed intakes of proteins, separating results into animal and plant proteins. Then, the protein contents in diets modeled with model set #2 (i.e., diets with progressive 5% step reductions in animal protein contributions to total proteins that are compatible with the fulfillment of all nutrient-based recommendations, except the recommendation on proteins, which was analyzed as output, at no additional cost and taking eating habits into account) were reported and compared to recommended levels, separating results into animal and plant proteins. Finally, for each subpopulation, the food content of the model set #2 selected diet (i.e., among the model set #2 diets, the diet with the lowest animal protein contribution to total proteins, compatible with nutrient and protein adequacy, affordability, and eating habits) was compared to the same food content for the observed diet.

## Results

**Table 2** shows the total and animal protein contents in the mean observed diets and in diets obtained with model set #1, for each subpopulation.

**TABLE 2:** Recommended protein intakes, total protein contents, and animal protein contents in OBS and in diets obtained with MOD1, for each subpopulation^1^

		Minimum recommended intake of total proteins,^2^ g/d	Total proteins				Animal proteins			
	Mean body weight, kg		In g/d		In % energy		In g/d		In % of total proteins	
Subpopulation			OBS	MOD1	OBS	MOD1	OBS	MOD1	OBS	MOD1
Women < 50 y	62.6	52.0	69.5	54.3	16.0	12.5	49.3	27.6	71.0	50.8
Women 50–64 y	67.7	56.2	71.5	51.0	16.7	11.9	51.2	27.2	71.6	53.3
Women ≥ 65 y	65.5	65.5	68.2	51.6	16.3	12.3	46.9	30.3	68.7	58.8
Men < 65 y	77.7	64.5	93.9	48.4	17.0	8.8	66.5	22.4	70.8	46.3
Men ≥ 65 y	78.8	78.8	88.0	48.0	17.0	9.2	59.6	24.7	67.8	51.4

1 MOD1 focused on determining the theoretical minimum level of total dietary proteins compatible with the fulfillment of all nutrient-based recommendations (without imposing a minimum amount of total proteins), without changing the total energy content and at no additional cost. Abbreviations: MOD1, model set #1; OBS, observed diets.

2 The recommended intake was estimated by multiplying the average body weight by the recommended intake, expressed in g/kg of body weight.

In the observed diets, regardless of the subpopulation, the total protein contents were higher than the minimum recommended protein intake. By construction, the protein contents in all diets obtained with model set #1 are the minimum protein quantities that would theoretically be compatible with meeting all other nutrient recommendations, for each subpopulation. Regardless of the subpopulation, that minimum protein quantity was more or less equal to 50 g/d, well below observed intakes (around 70 g/d and 90 g/d in women and men, respectively) and also below the recommended protein intake, except for the subpopulation of women < 50 y.

In the observed diets, regardless of the subpopulation, the protein contributions to total energy were between 16% and 17%. In contrast, in the model set #1 diets, the protein contributions to total energy dropped to between 12.5% (in women < 50 y old) and 8.8% (in men < 65 y old). In the observed diets, the animal protein contributions to total proteins were around 68% to 72%, and they decreased to percentages between 46.3% (in men < 65 years old) and 58.8% (in women ≥ 65 years old) in model set #1 diets.

**Supplemental Table 3** shows nutrient contents, costs, and fish contents in observed diets and model set #1 diets for the 5 subpopulations. Some values were exactly equal to values imposed by their respective (minimum or maximum) constraints. Such constraints are named “binding” or “active” constraints. Identifying them helps to point out which constraints are more difficult to fulfil than others. Binding constraints are also the ones with the greatest influence on the kinds and amounts of food introduced in modeled diets (i.e., the selections or “food choices” made by the models). Constraints to energy (equality), water (equality), SFA (maximum), fiber (minimum), zinc (minimum), and vitamin D (minimum) were binding for all 5 subpopulations, as were constraints to the diet cost (maximum) and fish content (maximum). Minimum constraints to alpha-linolenic acids and to calcium were binding for almost all subpopulations. Many other constraints were binding for some but not all subpopulations. Thus, the minimum constraint to iodine was binding in all diets modeled for women, but not for men. Conversely, constraints to total sugars (maximum) and magnesium (minimum) were binding in all diets modeled for men, but not for women. The constraint to iron was binding only in the subpopulation of women < 50 years old.

**FIGURE 1:**
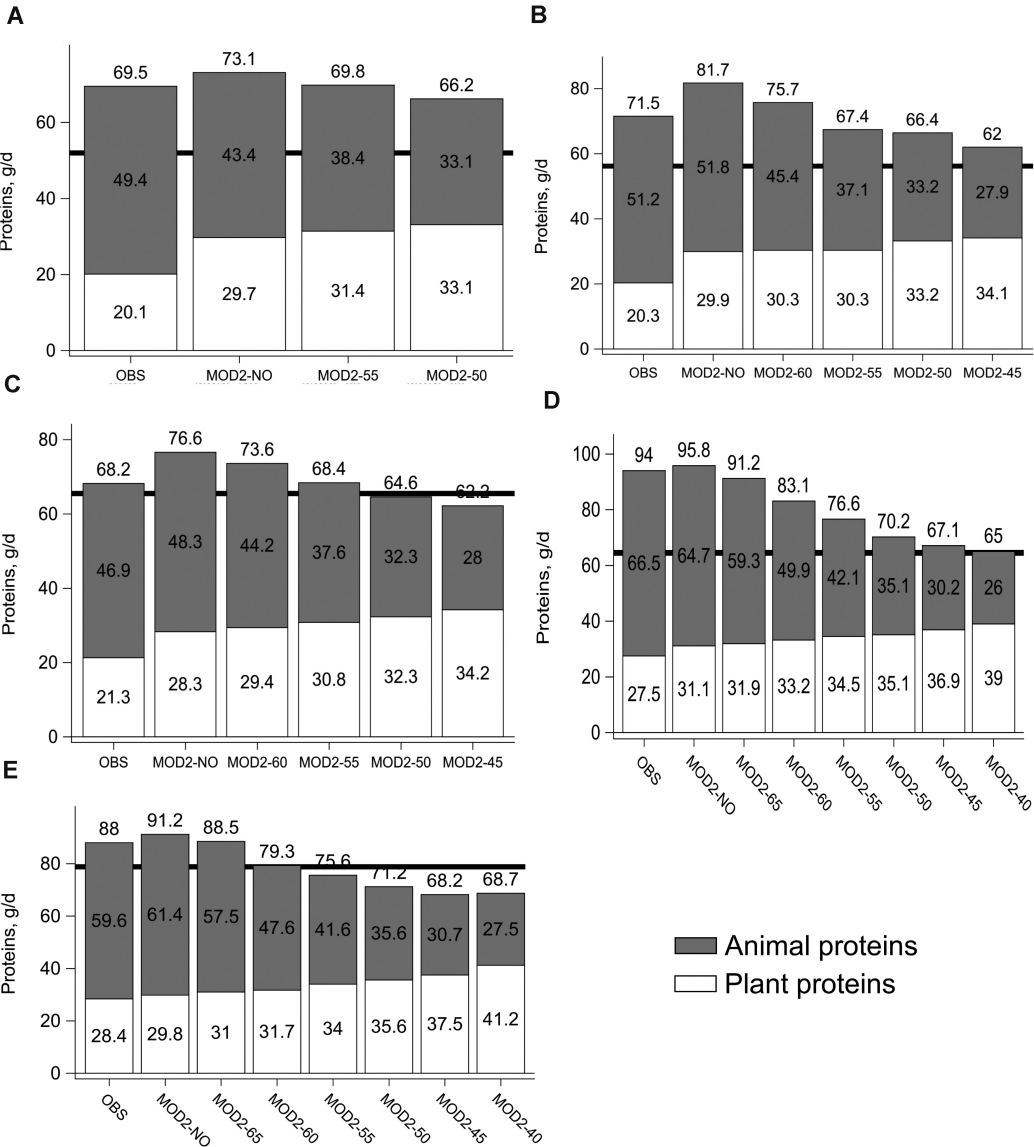
Animal proteins, plant proteins, and total protein contents (g/d) in OBS and in diets obtained with MOD2, MOD2-NO, and a progressively decreasing constraint (in 5% steps) on the animal protein contribution to total proteins^2^, until no solution could be found, in (A) women < 50 y, (B) women 50–64 y, (C) women ≥ 65 y, (D) men < 65 y, and (E) men ≥ 65 y. MOD2 imposed the fulfillment of all nutrient-based recommendations, except the recommendation for proteins, while minimizing the departure from the observed diet. MOD2-NO was a MOD2 model without constraints on animal protein contributions to total proteins. As an example of the decreasing constraints, MOD2-55 imposed a maximum animal protein contribution of 55% of the total proteins. The horizontal black line indicates the protein intake level recommended for the subpopulation. Abbreviations: MOD2, model set #2; MOD2-NO, without model set #2; OBS, observed diets.

**Figure 1** shows the protein contents of observed diets and diets obtained with model set #2. In an initial step, and compared to observed diets, fulfilling all the applied constraints increased the total protein content for all subpopulations, whereas no constraint was applied to proteins (model set #2-NO diets). Then, imposing a decrease in the animal protein contribution to total proteins (other diets in model set #2) induced a progressive decrease in the total protein content.

In most modeled diets, as shown by the bars exceeding the horizontal black line, the recommended level of protein intake was achieved despite the absence of a constraint applied to it. However, for older subpopulations, the modeled diets did not contain the recommended protein levels when the animal protein contributions to total proteins were ≤55% for men ≥ 65 y and ≤50% for women ≥ 65 y. In other subpopulations, the modeled diets fulfilled all nutritional constraints, and their protein contents were higher than the recommended levels (despite no constraints applied to them), with animal protein contributions to total proteins as low as 40% (men < 65 y), 45% (women 50–64 y), and 50% (women < 50 y). The cost constraint was binding in all the modeled diets (data not shown). All diets with total protein contents higher than the recommended levels were qualitatively and quantitatively adequate in terms of their amino acid contents, so that full protein adequacy was ensured (**Supplemental Table 4**).

**Supplemental Figure 1** shows the departures from the observed diets (i.e., the value, D, of the objective function; that is, the sum of the absolute differences between the quantity of each food in the observed diet and the corresponding modeled diet), as well as the numbers of remaining food items, induced by all model set #2 diets. Depending on the subpopulations, departures between 1.0 kg/d and 1.8 kg/d were needed to obtain the model set #2-NO diets. Then, a progressively decreasing animal protein contribution to total proteins increased the departure from the observed diet and decreased the number of foods in the modeled diet. The subpopulation's specificities were observed as follows:

1. For women < 50 y, imposing a 50% animal protein contribution to total proteins induced a departure (D) from the observed diet that was higher than the total mass of the observed diet.
2. For women 50–64 y, imposing a 45% animal protein contribution to total proteins induced a departure (D) from the observed diet (2.8 kg/d) that was higher than the total mass of the observed diet (2.5 kg/d).
3. For women ≥ 65 y, the modeled diet with a 50% animal protein contribution to total proteins did not reach the recommended protein level.
4. For men < 65 y, the departure from the observed diet was never higher than the total mass of the observed diet, and the protein contents in modeled diets were always higher than the recommended level. However, the number of food items dramatically decreased (−17%) between the modeled diets with 45% and 40% animal protein contributions to total proteins.
5. For men ≥ 65 y, the modeled diet with a 55% animal protein contribution to total proteins did not reach the recommended protein level (squares).

**FIGURE 2:**
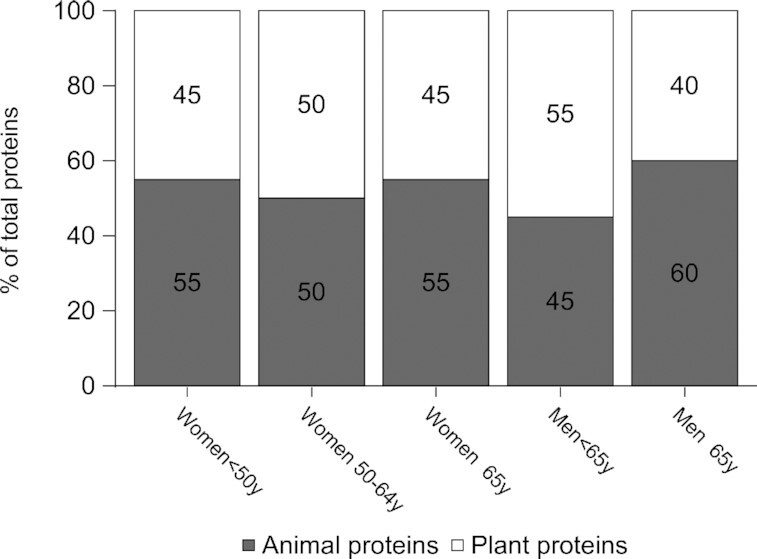
Animal and plant protein contributions to total proteins (in percentages) in the model set #2 selected diets (i.e., modeled diets with the lowest animal contributions to total proteins that are compatible with nutrient and protein adequacy, affordability, and eating habits) for each subpopulation. The models imposed the fulfillment of all nutrient-based recommendations except for the recommendation for proteins, while minimizing the departure from the observed diet.

Based on these results and the criteria described above to select 1 model set #2 diet per subpopulation, modeled diets with the following percentages of animal proteins in total proteins were selected for further analysis (**Figure 2**): 55% for women < 50 y, 50% for women 50–64 y, 55% for women ≥ 65 y, 45% for men < 65 y, and 60% for men ≥ 65 y.

**FIGURE 3:**
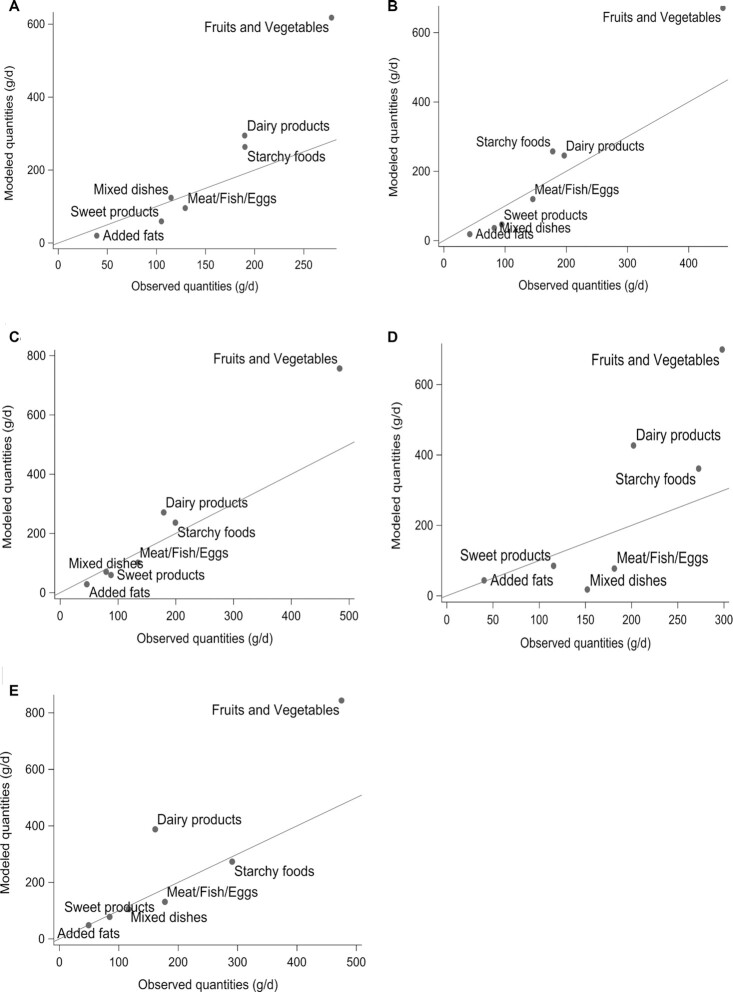
Observed (x-axis) and modeled (y-axis) quantities from selected MOD2 diets of each food group for each subpopulation. For (A) women < 50 y and (C) women ≥ 65 y, the MOD2 selected diets contained animal proteins as 55% of total proteins; for (B) women 50–64 y, the MOD2 selected diet contained animal proteins as 50% of total proteins; for (D) men < 65 y, the MOD2 selected diet contained animal proteins as 45% of total proteins; and for (E) men ≥ 65 y, the MOD2 selected diet contained animal proteins as 60% of total proteins. Abbreviation: MOD2, model set #2.

**Figure 3** shows the dietary shifts in food groups induced by each selected model set #2 diet, and **Table 3** displays the dietary shifts induced by the selected models in more detail. “Water and drinks” and “plant-based alternatives” were removed from the graph for visualization purposes because they presented quantities that were too high and too low, respectively, but were included in Table 3. In all selected modeled diets, fruits and vegetables, dairy products, and starchy foods increased (except the latter in men ≥ 65 y), while other food groups decreased.

**TABLE 3:** Group and subgroup quantities in OBS and MOD2 selected diets for each subpopulation^1^

	Women < 50 y		Women 50–64 y		Women ≥ 65 y		Men < 65 y		Men ≥ 65 y	
Food groups and subgroups, g/d	OBS	MOD2-55%^2^	OBS	MOD2-50%^2^	OBS	MOD2-55%^2^	OBS	MOD2-45%^2^	OBS	MOD2-60%^2^
Fruit and vegetables	278.3	617.7	455.8	671.3	483.5	756.6	298.1	699.6	475.1	844.0
Fruits (without juice)	112.5	300.7	202.9	352.7	203.0	351.9	122.5	327.0	200.3	432.9
Nuts and seeds	1.58	2.86	2.64	6.79	2.52	13.33	2.74	2.86	1.69	2.50
Vegetables	164.2	314.1	250.2	311.9	278.1	391.4	172.8	369.8	273.1	408.5
Starchy foods	190.1	263.3	177.8	257.3	199.3	236.3	272.5	361.3	290.9	273.3
Unrefined^3^	63.40	163.6	62.90	155.71	67.76	164.3	78.49	190.0	86.66	160.6
Refined	121.1	97.10	111.0	99.15	129.3	71.23	189.2	166.4	202.6	111.2
Breakfast cereals^4^	5.59	2.59	3.86	2.44	2.27	0.78	4.89	4.89	1.71	1.50
Dairy products	189.9	294.7	196.5	245.4	179.1	271.0	202.0	426.8	161.3	388.0
Milk	90.01	220.9	62.56	157.4	79.71	194.8	98.18	369.2	60.05	321.4
Yogurt	76.28	73.76	109.5	88.00	73.64	72.31	68.47	57.61	59.49	59.49
Cheese	23.57	0.00	24.50	0.00	25.74	3.87	35.41	0.00	41.80	7.10
Meat, fish, and eggs	129.3	95.61	145.4	119.9	134.5	101.3	181.3	77.41	177.6	131.4
Meat and deli meat	89.41	23.55	94.87	45.58	84.61	29.27	138.0	45.07	123.9	58.18
Fish	26.84	28.57	35.42	28.57	34.75	28.57	29.75	28.57	34.24	28.57
Eggs	13.05	43.49	15.11	45.71	15.15	43.49	13.56	3.77	19.45	44.64
Mixed dishes	114.9	124.0	82.30	36.41	79.53	70.66	152.1	18.01	116.6	105.3
Animal-based dishes^5^	89.59	124.0	58.75	36.02	48.23	69.02	123.1	11.37	68.01	57.90
Plant-based dishes^6^	25.27	0.00	23.55	0.39	31.31	1.64	29.03	6.63	48.58	47.36
Sweet products	105.1	59.43	94.79	46.48	87.96	59.81	115.6	85.20	84.61	78.02
Biscuits and sugar	32.53	12.02	29.52	8.20	24.17	10.60	35.71	0.15	26.79	2.77
Desserts	15.36	10.11	12.30	8.25	17.59	14.71	16.91	6.53	11.55	10.14
Cakes and tarts	57.19	37.30	52.97	30.02	46.20	34.50	62.97	78.52	46.27	65.11
Plant-based alternatives	3.07	2.79	9.38	9.38	6.99	6.99	2.43	2.43	1.49	1.49
Alternatives to meat	0.28	0.00	0.45	0.45	0.00	0.00	0.07	0.07	0.19	0.19
Alternatives to dairy^4^	2.79	2.79	8.93	8.93	6.99	6.99	2.36	2.36	1.30	1.30
Added fats	39.22	20.14	42.35	18.69	46.11	28.68	40.35	44.02	49.25	48.66
Animal fats	12.00	0.00	11.18	0.00	12.46	0.00	12.76	8.52	13.91	13.91
Vegetable fats	18.95	17.29	24.64	16.94	27.43	15.82	19.06	32.47	28.81	32.31
Spices and sauces	8.26	2.85	6.53	1.75	6.22	12.86	8.53	3.04	6.52	2.44
Waters and drinks	1305	900.6	1336	969.4	1157	836.2	1265	1265	1086	1086
Water	790.4	491.0	804.1	476.0	757.2	459.3	775.1	775.1	685.8	685.8
Sugary drinks	85.51	0.00	20.02	0.00	9.48	0.00	109.2	109.2	15.60	15.60
Fruit juices (100%)	60.88	41.03	48.37	30.18	31.59	17.77	57.39	57.39	41.26	41.26
Tea and coffee	368.5	368.5	463.2	463.2	359.1	359.1	323.3	323.3	343.0	343.0

1 For each subpopulation, the selected diet is the diet, among the MOD2 diets, with the lowest animal protein contribution (in percentage of total proteins) fulfilling all nutrient-based recommendations, including the recommendation on proteins, without changing the energy content, at no additional cost, and taking eating habits into account. Abbreviations: MOD2, model set #2; OBS, observed diets.

2 Numbers for the MOD2 diets indicate the animal protein percentages of the total protein in the selected modeled diet.

3 Including legumes (lentils, white beans, etc.).

4 Quantities of all fortified foods (e.g., breakfast cereals or plant-based alternatives to dairy) were constrained to not increase compared to observed amounts.

5 Including animal-based salted tarts (such as quiches), sandwiches, burgers, couscous, paella, and so forth.

6 Including plant-based salted mixed dishes (such as tabouleh) and appetizers or biscuits.

All subgroups within the fruit and vegetable food group had increased intakes between the observed and modeled diets, as did unrefined starchy foods, milk, and eggs (except the latter in men < 65 y). In comparison, intakes of refined starchy foods, breakfast cereals (except in men < 65 y), yogurt (except in men ≥ 65 y), cheese, meat and deli meat, plant-based dishes, biscuits and sugar, desserts, animal fats (except in men ≥ 65 y), and spices and sauces (except in women ≥ 65 y) decreased. Fish intakes attained the maximum applied amount (200 g per week). Tea and coffee intakes remained constant. Other subgroups from the water and drinks group had decreased intakes for women and remained constant for men. For other food subgroups (animal-based dishes, cakes and tarts, and vegetable fats), the directions of variation differed between subpopulations.

## Discussion

It is known that foods that are sources of protein contain several nutrients other than protein but, to the best of our knowledge, this study is the first that assessed to what extent total proteins and animal protein contributions to total proteins could be theoretically reduced without impairing the fulfillment of all other nutrient-based recommendations, excluding the use of nutritional supplements or fortified foods. A mathematical optimization approach was used to model population diets while simultaneously fulfilling a set of nutrient-based constraints (not including the constraint on proteins), without changing dietary energy and at no additional cost. The results showed that in highly theoretical models where total proteins were directly minimized without considering eating habits, a strict minimum of at least 48 g/d of total proteins was needed to meet nutrient-based recommendations for nutrients other than proteins. Models better at taking eating habits into account showed that the animal protein contributions to total proteins, which were approximately 70% in observed diets, could be reduced to contributions that were between 45% and 60%, depending on age and sex, while still being compatible with complete nutritional adequacy and affordability. Lower percentages would either be mathematically unattainable or constraints would have to be relaxed or removed, therefore impairing nutritional adequacy and/or realism in the resulting modeled diets.

One important finding from this study is that in the absence of any constraint imposing a given protein level, a strict minimum protein amount was needed to cover other nutrient-based recommendations in adults (model set #1). This quantity was close to the minimum recommended protein intake level for each subpopulation. For the subpopulation of women < 50 y (where the recommended protein level was relatively low due to their low average body weight), the strict minimum was even higher than the recommended protein level. Numerous nutritional constraints were binding in model set #1, showing that foods that are a source of protein were needed for several other nutrients. Achieving the recommended levels was difficult for vitamin D, zinc, fiber, alpha-linolenic acid, and SFA in all subpopulations and for iodine, calcium, magnesium, and total sugars in some subpopulations. Regarding iron, the constraint was binding only for young women. In that subpopulation, the level of the iron constraint (i.e., 16 mg/d) imposed corresponded to a hypothesis of high iron losses in menstrual blood. Note that alternative models (data not shown) were conducted where only 11 mg/d of iron (recommended iron intake for young women with normal iron losses) was required, and the minimized total protein contents did not change (54 g).

Despite the absence of any constraint related to amino acid contents in diets, indispensable amino acids were systematically adequate in all modeled diets fulfilling nutrient recommendations, including the recommendation for total proteins, whether their animal protein contribution to total proteins was decreased or not. This means that other nutrient deficiencies would occur before amino acid deficiency would become a problem. Accordingly, previous studies showed that protein adequacy is slightly or not influenced by the amino acid distributions in foods that are a source of protein (4), and is more significantly related to protein quantity than quality, except in diets containing high plant protein contributions to total proteins (around 70%) from minimally diversified sources, such as refined grains (20).

Foods that were sources of both animal and plant proteins were needed to cover nutrient requirements, even when proteins were minimized. Shifting towards more plant-based diets in Western countries is often recommended by the scientific community and public stakeholders for health and climate purposes (1,21–23), but there is no consensus regarding the adequate ratio of animal protein to plant protein in sustainable diets. A 1:1 ratio (1 g of plant protein for 1 g of animal protein) is often presented as a nutritional standard but, to the best of our knowledge, it is not recommended by any official order. In this study, models decreasing the animal protein contributions to total proteins by 5% steps (model set #2) showed that animal protein contributions of 60% (men ≥ 65 y), 55% (women < 50 y and ≥ 65 y), 50% (women 50–64 y), and 45% (men < 50 y) would be fully compatible with adequate nutrients and proteins, affordability, and eating habits, despite their relatively low protein contents. Our results therefore imply that moving towards diets with lower animal protein contributions or to fully vegan diets, like the ones included within the range of diets recommended by the EAT–Lancet Commission (21), would necessarily require food fortification and/or nutrient supplementation to cover adult nutritional requirements. The minimum percentages of animal proteins found in the present study to be compatible with nutrient adequacy in the different subpopulations considered can be compared to those obtained in 2 recent studies focused on designing sustainable diets: 1 for French adults from the NutriNet-Santé cohort (24) and 1 for older Dutch adults from the Longitudinal Aging Study Amsterdam cohort (25). In apparent contradiction to our results, for the French study, the mathematical optimization approach used to derive nutritionally adequate individual diets with progressively lower environmental impacts, while also controlling for costs, resulted in diets with an animal protein contribution to total protein as low as 22% for the most so-called “disruptive” scenario (24). Such a discrepancy can be explained through several methodological differences. Unlike our study, the NutriNet-Santé study did not consider vitamin D and omega 3 fatty acids; fiber was set at a minimum level of only 23 g/d (compared to 30 g/d, the French recommendation, in our study); fortified foods appeared to be allowed to increase (whereas they were constrained to not increase in our study); the minimum constraint on total proteins was set at only 0.66 g/kg of body weight, without any age distinction; and the amino acid content was not analyzed. As suggested by the term “disruptive,” a considerable departure from observed food consumption patterns was allowed, and 200 g/d of soya-based products was included (with no information on possible fortification). Results from the Dutch study were more in line with our results, since they showed that it was possible to design a high-protein (defined as providing > 1.2 g/kg body weight), nutritionally adequate diet with a lower climate impact, a 50% contribution in animal protein, and lower contributions that induce amino acid inadequacy (25). This is lower than the 60% and 55% contributions we obtained for older men and women, respectively, but fewer nutritional constraints were included in the Dutch study than in our models.

Regardless of the subpopulation, all modeled diets reached the maximum allowable diet cost (i.e., the observed cost), confirming that reaching nutritional adequacy tends to be costly (12). The presence of both animal- and plant-based food sources in those diets is in line with several studies showing that low-cost, nutritionally adequate diets, even highly theoretical ones, always include animal-based foods (26, 27). In this study, regardless of the subpopulation, milk, eggs, and unrefined starches were increased in all modeled diets selected, probably because they are affordable sources of nutrients (28). To the contrary, foods with inadequate nutrient densities (biscuits and sugar, for example) or that are nutrient dense but more expensive than other foods with similar characteristics (cheese, for example) were decreased or removed.

This study presents some limitations. The modeled diets are theoretical and were not assessed for their acceptance within the population. Results that were expressed in terms of food amounts in selected modeled diets show large differences from what is currently consumed (between +47% and +135% of fruits and vegetables, for example), which are not likely to be adopted in the short term. Nevertheless, diet optimization is a powerful approach to simultaneously consider demands on several metrics in order to identify dietary shifts that are able to improve health and food consumption sustainability (29, 30). Studies are integrating more and more enhancements, such as considering coproduction links between food items (e.g., milk and beef), price elasticities, environmental impacts, food contaminants, or nutrient bioavailability (29). In this study, bioavailability was considered for zinc (recommended levels adapted to phytate levels), iron (assumptions on high iron losses and related iron needs), and protein (quantitative and qualitative amino acid assessments). The presence of food contaminants was only partially and indirectly taken into account, by imposing a maximum quantity of fish. Note, however, that any additional constraints applied to the models would have had no impact on the results (in the case of inactive constraints) or would have increased the minimum total protein quantities (model set #1) or the minimum animal protein contributions to total proteins (model set #2) in modeled diets (in the case of active constraints). Another limitation is that data were limited to the French adult population and were not the most recent data available. It would be interesting to replicate the approach using the most recent data and other populations, especially children and populations from countries with differing protein intake patterns (31).

In conclusion, this study showed that for this French adult population, the lowest animal protein contributions to total proteins that are compatible with nutritional adequacy, affordability, and eating habits vary from 45% to 60%, depending on age and sex, with the highest contributions needed for older populations and young women. The environmental impacts from resulting dietary shifts need to be assessed in order to verify their benefits to planetary health.

## Supplementary Material

nxac150_Supplemental_File

## Notes

MS-Nutrition and MoISA received financial support from the French National Interprofessional Association of Livestock and Meat (Interbev).

Author disclosures: The authors report no conflicts of interest.

Interbev had no role in the design, implementation, analysis, or interpretation of the data.

Supplemental Figure 1 and Supplemental Tables 1–4 are available from the “Supplementary data” link in the online posting of the article and from the same link in the online table of contents at http://jn.nutrition.org.

Abbreviations used: INCA2, Second French Individual and National Study on Food Consumption; NRV, nutrient reference values.
