# Supplementary material for: Approximately Half of Total Protein Intake by Adults Must be Animal-Based to Meet Nonprotein, Nutrient-Based Recommendations, With Variations Due to Age and Sex
Source: J Nutr. 2022 Jul 11;152(11):2514–25. doi: 10.1093/jn/nxac150 (PMC9644177; doi:10.1093/jn/nxac150)
Supplement: nxac150_Supplemental_File [file nxac150_supplemental_file.docx]

**Approximately half of total protein intake by adults must be animal-based to meet non-protein nutrient-based recommendations with variation due to age and sex.** Vieux et al. Online Supplementary Material

**Supplemental Table 1. Food hierarchy of the 212 frequently consumed food items and main source of proteins**

| Food group | Food subgroup | Food category | Food item | Main source of proteins* |
| --- | --- | --- | --- | --- |
| Fruit and Vegetables | Vegetables, raw, cooked and soups | Cooked vegetables | Tomato sauce. without meat | VEG |
|  |  |  | Beans. mung. sprouted. canned. drained | VEG |
|  |  |  | Ratatouille | BOTH |
|  |  |  | Celery stalk. boiled | VEG |
|  |  |  | Cauliflower. boiled | VEG |
|  |  |  | Courgette (zucchini). boiled. with skin | VEG |
|  |  |  | Spinach. steamed | VEG |
|  |  |  | Green bean. canned. drained | VEG |
|  |  |  | Sweet corn. canned | VEG |
|  |  |  | Turnip. boiled | VEG |
|  |  |  | Onion. boiled | VEG |
|  |  |  | Pea. green. canned | VEG |
|  |  |  | Leek. boiled | VEG |
|  |  |  | Pepper. sweet. green or red. boiled | VEG |
|  |  |  | Artichoke. globe. boiled | VEG |
|  |  | Raw vegetables | Cucumber. peeled. raw | VEG |
|  |  |  | Radish. raw | VEG |
|  |  |  | Carrot. raw | VEG |
|  |  |  | Celeriac. raw | VEG |
|  |  |  | Witlof. raw | VEG |
|  |  |  | Green salad. without seasoning | VEG |
|  |  |  | Tomato. raw | VEG |
|  |  | Vegetable soups | Soup. vegetable. canned | VEG |
|  |  |  | Soup. cream of tomato | BOTH |
|  |  |  | Soup. vegetable (average) | VEG |
|  | Fresh and processed fruits (excl. dried) | Fresh fruits | Apricot. raw | VEG |
|  |  |  | Banana. pulp. raw | VEG |
|  |  |  | Cherry. raw | VEG |
|  |  |  | Strawberry. raw | VEG |
|  |  |  | Kiwi fruit. pulp and seeds. raw | VEG |
|  |  |  | Clementine or Mandarin orange. pulp. raw | VEG |
|  |  |  | Melon. raw | VEG |
|  |  |  | Orange. sweet. pulp. raw | VEG |
|  |  |  | Pear. flesh and skin. raw | VEG |
|  |  |  | Apple. with skin. raw | VEG |
|  |  |  | Grapefruit. pulp. raw | VEG |
|  |  |  | Grape. white. raw | VEG |
|  |  |  | Peach. flesh and skin. raw | VEG |
|  |  | Processed fruits | Applesauce. canned. sweetened | VEG |
|  |  |  | Stewed fruits. canned | NO |
|  |  |  | Stewed fruits. low calorie. canned | VEG |
|  |  |  | Fruit cocktail. canned | VEG |
|  | Dried fruits and oilseeds | Oilseeds | Walnut | VEG |
|  |  |  | Prune | VEG |
| Starchy foods | Refined starches | Bread | Bread. plain. toasted at home | VEG |
|  |  |  | Bread. sandwich loaf | BOTH |
|  |  |  | Rusk. plain | VEG |
|  |  |  | Bread. French 250 g baguette. from bakery | VEG |
|  |  |  | Bread. French. country style. from bakery | VEG |
|  |  | Pasta, rice, semolina | Couscous grains. cooked | VEG |
|  |  |  | Pasta. egg. boiled | BOTH |
|  |  |  | Pasta. boiled | VEG |
|  |  |  | Rice. white. boiled | VEG |
|  | Unrefined starches | Legumes | Haricot bean. boiled | VEG |
|  |  |  | Lentil. boiled | VEG |
|  |  | Wholemeal products | Bread. wholemeal. from bakery | VEG |
|  |  |  | Bread. mixed cereal. from bakery | VEG |
|  |  |  | Wheat. whole grain. plain. boiled | VEG |
|  |  | Potatoes | Mashed potatoes. prepared with milk and butter. not salted | BOTH |
|  |  |  | Potato. boiled | VEG |
|  |  |  | Potatoes. pan-fried. frozen. not salted | VEG |
|  | Breakfast cereals | Breakfast cereals | Breakfast cereals. wheat flakes. chocolate. enriched | BOTH |
|  |  |  | Muesli (average) | VEG |
|  |  |  | Breakfast cereals. corn flakes. plain. enriched | VEG |
| Dairy products | Milk | Plain milk | Milk. semi-skimmed. UHT | ANI |
|  |  |  | Milk. skimmed. UHT | ANI |
|  |  |  | Milk. whole. UHT | ANI |
|  |  | Sweetened milk | Chocolate flavoured beverage. sweetened. prepared | BOTH |
|  | Yoghurt | Plain yoghurt | Quark 20% fidm. plain | ANI |
|  |  |  | Yoghurt. wholemilk. Bifidus. plain | BOTH |
|  |  |  | Yoghurt. plain (average) | ANI |
|  |  | Sweetened or flavored yoghurt | Drinking yoghurt with L. Casei. lowfat. plain | ANI |
|  |  |  | Yoghurt. nonfat. with fruit. aspartame | ANI |
|  | Cheese | Cheese | Roquefort cheese | ANI |
|  |  |  | Bonbel-Babybel ® type cheese | ANI |
|  |  |  | Processed cheese 45% fidm | ANI |
|  |  |  | Camembert 45% fidm | ANI |
|  |  |  | Tomme cheese | ANI |
|  |  |  | Goat cheese from raw or pasteurised milk | ANI |
|  |  |  | Mimolette cheese | ANI |
|  |  |  | Gruyere cheese | ANI |
| Meat/Fish/Eggs | Meat and deli meat | Red meat | Lamb leg. roasted | ANI |
|  |  |  | Beef. ground. 15% fat. cooked | ANI |
|  |  |  | Pork chop. grilled | ANI |
|  |  |  | Pork roast. lean & fat. cooked | ANI |
|  |  |  | Veal cutlet. cooked | ANI |
|  |  |  | Ham. cooked | ANI |
|  |  | Poultry and game | Turkey. meat only. roasted | ANI |
|  |  |  | Turkey. breast. meat only. sauteed | ANI |
|  |  |  | Duck. magret. cooked in pan | ANI |
|  |  |  | Chicken. meat & skin. roasted | ANI |
|  |  | Deli meat | Foie gras. canned | ANI |
|  |  |  | Merguez sausage. beef & mutton. cooked | ANI |
|  |  |  | Strasbourg sausage | ANI |
|  |  |  | Pork rashers. lean. cooked | ANI |
|  |  |  | Chipolata sausage. cooked | ANI |
|  |  |  | Ham. raw. cured | ANI |
|  |  |  | Dry sausage | ANI |
|  |  |  | Pâté. country style | BOTH |
|  |  | Offals | Liver. calf. cooked | ANI |
|  | Fish | Lean fish | Atlantic pollock. cooked | ANI |
|  |  |  | Fish cakes. fried | ANI |
|  |  |  | Tuna. canned in brine. drained | ANI |
|  |  | Seafoods | Scallop. steamed | ANI |
|  |  |  | Shrimp or prawn. boiled | ANI |
|  |  |  | Oyster. raw | ANI |
|  |  |  | Mussel. boiled | ANI |
|  |  | Fatty fish | Salmon. smoked | ANI |
|  |  |  | Salmon. steamed | ANI |
|  |  |  | Tuna. oven cooked | ANI |
|  | Eggs | Eggs | Egg. hard-boiled | ANI |
|  |  |  | Omelet. plain | BOTH |
| Mixed dishes | Meat-based dishes | Animal-based composite dishes | Cassoulet. canned | BOTH |
|  |  |  | Shepherd's pie | BOTH |
|  |  |  | Pot-au-feu | ANI |
|  |  |  | Couscous with mutton and beef | BOTH |
|  |  |  | Paëlla | BOTH |
|  |  |  | Poultry filled with cheese and ham | ANI |
|  |  |  | Ravioli with meat and tomato sauce. canned | BOTH |
|  |  | Animal-based sandwich, burger, pizza | Pizza (average) | BOTH |
|  |  |  | Cheeseburger. fast foods restaurant | BOTH |
|  |  |  | Sandwich on french bread. ham and butter | BOTH |
|  |  | Salted tarts, fried specialities and related | Quiche lorraine | BOTH |
|  |  |  | Crepe. stuffed with ham and cheese | BOTH |
|  | Plant-based dishes | Plant-based composite dishes | Sauerkraut canned | BOTH |
|  |  |  | Tabouleh. commercial | VEG |
|  |  | Salty appetizer | Potato crisp (US=chip). salted | VEG |
|  |  |  | Cocktail biscuit without cheese | VEG |
|  |  | Stocks and soup broths | Soup. chicken noodle | BOTH |
| Sweet products | Biscuits and sugar | Biscuits and cereals bars | Biscuit (cookie). boat-shaped. jam filled. commercial | BOTH |
|  |  |  | Chocolate candy bar. biscuit-caramel filling | BOTH |
|  |  |  | Biscuit (cookie). snack with chocolate filling | BOTH |
|  |  |  | Biscuit (cookie) | BOTH |
|  |  | Sweets and chocolate | Fruit syrup | NO |
|  |  |  | Sweets (candy) | BOTH |
|  |  |  | Chocolate. milk- | BOTH |
|  |  |  | Chocolate. milk-. with nuts and raisins | BOTH |
|  |  |  | Chocolate. dark-. 40% cocoa | VEG |
|  |  | Honey, jam, spreads | Beverage base. chocolat flavoured. sweetened (powder) | VEG |
|  |  |  | Honey | VEG |
|  |  |  | Chocolate paste with hazelnuts | BOTH |
|  |  |  | Jam or marmalade | VEG |
|  |  | Sugar | Sugar. white | NO |
|  | Desserts | Desserts | Chocolate mousse | BOTH |
|  |  |  | Chocolate custard. topped with whipped cream | BOTH |
|  |  |  | Custard with caramel sauce | ANI |
|  |  |  | Custard dessert. chocolate. refrigerated | BOTH |
|  | Cakes and tarts | Cakes, pies, custards, ice creams | Cake. chocolate | BOTH |
|  |  |  | Fruit cake | BOTH |
|  |  |  | Fruit batter pudding | BOTH |
|  |  |  | Flan with pie crust | BOTH |
|  |  |  | Mini pound cake with chocolate filling | BOTH |
|  |  |  | Madeleine biscuit (cookie) | BOTH |
|  |  |  | Eclair. from bakery | BOTH |
|  |  |  | Cake (average) | BOTH |
|  |  |  | Fruit pie or tart. from bakery | BOTH |
|  |  |  | Ice cream or sorbet or ice pop. flavoured (average) | BOTH |
|  |  | Viennoiseries | Croissant. with chocolate filling. from bakery | BOTH |
|  |  |  | Croissant. ordinary or butter | BOTH |
|  |  |  | Brioche. commercial | BOTH |
|  |  |  | Crepe with sugar | BOTH |
|  |  |  | Crepe. plain | BOTH |
| Plant-based alternatives | To meat | Plant protein products | Tofu. soy bean curd | VEG |
|  |  |  | Soy burger | VEG |
|  | To dairy | Yoghurt substitutes | Soy yoghurt. plain. refrigerated | VEG |
|  |  |  | Soy yoghurt with fruit. refrigerated | VEG |
|  |  |  | Soy dessert. flavoured. refrigerated | VEG |
|  |  | Vegetable drinks | Soya drink. plain | VEG |
| Added fats | Animal fats | Butter | Butter. unsalted | ANI |
|  |  |  | Low-fat butter | ANI |
|  |  |  | Butter. salted (0.5-3%) | ANI |
|  |  | Cream | Cream (average) | ANI |
|  | Vegetable fats | Vegetable fat | Mayonnaise | BOTH |
|  |  |  | Salad dressing. oil & vinegar | NO |
|  |  | Oil | Oil. rapeseed | NO |
|  |  |  | Oil. olive | NO |
|  |  |  | Oil. soya | NO |
|  |  |  | Oil. sunflowerseed | NO |
|  |  |  | Oil. vegetable (average) | NO |
|  |  | Margarine | Margarine 80% fat. hard | NO |
|  |  |  | Fat spread 60% fat | BOTH |
|  | Spices and sauces | Spices | Soy sauce | VEG |
|  |  | Sauces | Ketchup | VEG |
|  |  |  | Tomato sauce. with meat | BOTH |
| Water and drinks | Water | Tap water | Water. municipal | NO |
|  |  | Mineral water | Water. bottled (average) | NO |
|  |  |  | Water. mineral. non-carbonated. CONTREX | NO |
|  |  |  | Water. mineral. non-carbonated. EVIAN | NO |
|  |  |  | Water. mineral. non-carbonated. HEPAR | NO |
|  |  |  | Water. mineral. non-carbonated. VITTEL | NO |
|  |  |  | Water. mineral. non-carbonated. VOLVIC | NO |
|  |  |  | Water. mineral. carbonated. PERRIER | NO |
|  |  |  | Water. mineral. carbonated. BADOIT | NO |
|  | Sugary drinks | Sweetened drinks | Carbonated beverage. lemon or clear lime. sweetened | NO |
|  |  |  | Tea flavored beverage. sweetened | NO |
|  |  |  | Carbonated beverage. orange with pulp (14% fruit). sweetened | NO |
|  |  |  | Fruit still drink (10-50% juice). orange. sweetened | NO |
|  |  |  | Carbonated beverage. cola | NO |
|  | Fruit juices | Fruit juices | Pineapple juice. reconstituted. pasteurized | VEG |
|  |  |  | Fruit juice. mixed. enriched. pasteurized | VEG |
|  |  |  | Orange juice. reconstituted. pasteurized | VEG |
|  |  |  | Orange juice. raw. unsweetened | VEG |
|  |  |  | Apple juice. reconstituted. pasteurized | NO |
|  |  |  | Grape juice. pasteurized | NO |
|  | Tea and coffee | Coffee, tea and infusions | Coffee. black. unsweetened | NO |
|  |  |  | Coffee. instant. reconstituted | NO |
|  |  |  | Tea. unsweetened | NO |
|  | Alcoholic beverages | Alcoholic beverages | Anis spirit. diluted (1+5) | NO |
|  |  |  | Champagne | NO |
|  |  |  | Beer. regular (4-5° alcohol) | VEG |
|  |  |  | Cider (average) | NO |
|  |  |  | Wine. red 12° | NO |

***NO: trivial amount (≤0.3 g/100g) of proteins; VEG: more than 0.3g of proteins/100g and more than 95% of proteins content is plant based; ANI: more than 0.3g of proteins/100g and more than 95% of proteins content is animal based; BOTH otherwise**

**Supplemental Table 2. Reference for indispensable amino acid content in diets** (from ANSES-Rapport d’expertise collective. Actualisation des repères du PNNS: élaboration des références nutritionnelles. 2016)

| Indispensable amino acids | Reference (mg/g of proteins) |
| --- | --- |
| Histidine | 17 |
| Isoleucine | 27 |
| Leucine | 59 |
| Lysine | 45 |
| Methionine+Cysteine | 23 |
| Phenylalaline+tyrosine | 41 |
| Threonine | 25 |
| Tryptophan | 6 |
| Valine | 27 |

**Supplemental Table 3. Nutrient content, cost and fish content in observed diets (OBS) and in diets obtained with Models#1 (MOD1), for each subpopulation^1^**

|  |  | Women<50y | | Women 50-64y | | | Women≥65y | | Men<65y | | Men≥65y | |
| --- | --- | --- | --- | --- | --- | --- | --- | --- | --- | --- | --- | --- |
|  |  | OBS | MOD1 | OBS | MOD1 | OBS | | MOD1 | OBS | MOD1 | OBS | MOD1 |
| Energy (kcal/d) | =observed | 1732 | 1732 | 1711 | 1711 | 1675 | | 1675 | 2207 | 2207 | 2083 | 2083 |
| Carbohydrates (% energy) | [40-55] | 43.41 | 47.17 | 42.23 | 44.46 | 43.26 | | 42.89 | 43.56 | 47.64 | 43.15 | 47.02 |
| Fats (% energy) | [35;40] | 38.33 | 35.91 | 38.40 | 39.05 | 37.84 | | 40.00 | 37.26 | 40.00 | 37.44 | 40.00 |
| Linoleic acids (% energy) | ≥4 | 5.04 | 4.84 | 5.40 | 6.51 | 5.54 | | 7.28 | 4.70 | 8.12 | 5.29 | 7.89 |
| Alpha-linolenic acids (% energy) | ≥1 | 0.46 | 1.00 | 0.52 | 1.00 | 0.51 | | 1.00 | 0.44 | 1.00 | 0.49 | 1.01 |
| DHA+EPA (g/d) | ≥250 | 0.20 | 0.39 | 0.24 | 0.38 | 0.23 | | 0.38 | 0.24 | 0.25 | 0.28 | 0.25 |
| SFA (% energy) | <12 | 14.68 | 12.00 | 14.21 | 12.00 | 13.80 | | 12.00 | 14.43 | 12.00 | 13.94 | 12.00 |
| Lauric myristic palmitic (% energy) | ≤8 | 9.54 | 7.84 | 9.22 | 7.49 | 9.03 | | 7.34 | 9.39 | 8.00 | 9.12 | 7.87 |
| Total sugars (without lactose) (mg/d) | ≤100 | 49.18 | 67.18 | 52.13 | 93.33 | 49.38 | | 80.24 | 57.52 | 100.0 | 55.11 | 100.0 |
| Sodium (mg/d) | ≤Observed | 2613 | 2113 | 2625 | 1754 | 2689 | | 1755 | 3460 | 2645 | 3576 | 1906 |
| Sodium/Potassium ratio (in molar terms) | ≤1 | 1.80 | 1.00 | 1.64 | 0.71 | 1.78 | | 0.79 | 1.96 | 1.00 | 2.03 | 0.73 |
| Water (ml/d) | =2000 (Women) or =2500 (Men) | 1992 | 2000 | 2179 | 2000 | 2020 | | 2000 | 2065 | 2500 | 2002 | 2500 |
| Fiber (g/d) | ≥30 | 14.27 | 30.00 | 16.92 | 30.00 | 16.83 | | 30.00 | 17.90 | 30.00 | 20.00 | 30.00 |
| Vitamin A (µg/d) | [650;3000] (Women) or [750;3000] (Men) | 940.2 | 1823 | 1254.7 | 1660 | 1239.6 | | 2747 | 1270.0 | 2207 | 1693.2 | 2838 |
| Thiamin (mg/MJ) | ≥0.14 (Women<50 & Men<65) or ≥0.10 (others) | 1.00 | 1.04 | 1.03 | 0.93 | 0.98 | | 0.94 | 1.26 | 1.28 | 1.21 | 0.87 |
| Vitamin B12 (µg/d) | ≥4 | 4.94 | 5.97 | 6.24 | 4.23 | 5.83 | | 10.80 | 6.58 | 6.31 | 7.59 | 10.95 |
| Riboflavin (mg/MJ) | ≥0.17 | 1.40 | 1.50 | 1.52 | 1.56 | 1.42 | | 1.87 | 1.68 | 1.65 | 1.66 | 1.70 |
| Niacin (mg/d) | ≤900 | 15.87 | 17.30 | 16.89 | 15.56 | 15.89 | | 17.58 | 21.26 | 17.87 | 20.29 | 18.05 |
| Niacin (mg NE eq/MJ) | ≥1.6 mg/MJ | 2.19 | 2.39 | 2.36 | 2.17 | | 2.27 | 2.51 | 2.30 | 1.93 | 2.33 | 2.07 |
| Pantothenic acid (mg/d) | ≥4.7 (Women) or ≥5.8 (Men) | 4.26 | 5.51 | 4.52 | 5.67 | 4.28 | | 6.55 | 5.28 | 6.08 | 5.22 | 6.10 |
| Vitamin B6 (mg/d) | [1.5;25] (Women<50) or [1.6;25] (Women≥50 yo) or [1.8;25] (Men<65 yo) or 1.7 (Men≥65 yo) | 1.39 | 1.68 | 1.53 | 1.90 | 1.47 | | 1.84 | 1.76 | 2.26 | 1.80 | 2.14 |
| Folates (µg/d) | ≥330 | 237.4 | 443.1 | 272.1 | 546.2 | 264.7 | | 505.5 | 283.1 | 586.1 | 310.9 | 489.1 |
| Vitamin C (mg/d) | ≥110 | 78.22 | 168.2 | 98.30 | 198.3 | 101.8 | | 195.8 | 79.64 | 206.3 | 97.73 | 186.4 |
| Vitamin D (µg/d) | [5;50] | 2.20 | 5.00 | 2.50 | 5.00 | 2.31 | | 5.00 | 2.57 | 5.00 | 2.73 | 5.00 |
| Vitamin E (mg/d) | [9.9;300] (women) [10.5;300] (men) | 10.79 | 14.51 | 11.85 | 18.24 | 11.68 | | 16.03 | 11.62 | 19.79 | 13.32 | 19.35 |
| Calcium (mg/d) | [960;2500] (women<50 yo and Men<65 yo) or [950;2500] (others) | 745.8 | 959.4 | 810.8 | 950.0 | 742.3 | | 950.0 | 874.7 | 1053 | 819.7 | 950.0 |
| Copper (mg/d) | [1;5] (Women<50 yo) or [1.6;5] (Men≥65 yo) or [1.3;5] (others) | 1.30 | 2.08 | 1.52 | 1.79 | 1.43 | | 2.85 | 1.63 | 1.97 | 1.84 | 2.91 |
| Iron (mg/d) | ≥16 (women<50yo) or ≥11 (others) | 9.23 | 16.00 | 9.63 | 13.04 | 8.88 | | 13.18 | 11.62 | 13.56 | 11.28 | 12.41 |
| Iodine (µg/d) | [150;600] | 104.3 | 150.0 | 108.6 | 150.0 | 102.5 | | 150.0 | 122.6 | 193.8 | 117.6 | 178.6 |
| Magnesium (mg/d) | ≥360 (women) or ≥420 (men) | 265.6 | 378.3 | 296.3 | 398.7 | 265.9 | | 370.9 | 323.9 | 420.0 | 310.4 | 420.0 |
| Phosphorus (mg/d) | ≥700 (women<50 yo and men<65 yo) or ≥550 (others) | 1034 | 1141 | 1055 | 1080 | 1000 | | 1114 | 1305 | 1233 | 1210 | 1111 |
| Selenium (µg/d) | [70;300] | 72.05 | 70.00 | 83.17 | 70.00 | 74.73 | | 70.00 | 84.41 | 86.03 | 83.53 | 75.77 |
| Zinc (mg/d) | [7.5; 25]* | 8.38 | 9.17 | 8.87 | 8.06 | 8.17 | | 9.20 | 11.32 | 10.93 | 10.75 | 10.64 |
| Fish (g/d) | ≤28.57 | 25.89 | 28.57 | 34.21 | 28.57 | 33.33 | | 28.57 | 28.46 | 28.57 | 32.55 | 28.57 |
| Diet cost (euros/d) | ≤observed | 5.16 | 5.16 | 5.77 | 5.77 | 5.52 | | 5.52 | 6.33 | 6.33 | 6.30 | 6.30 |

^1^Models#1were aimed at determining the theoretical minimum level of total dietary proteins compatible with the fulfilment of all nutrient-based recommendations (without applying a minimum quantity of total proteins) without increasing total energy content and at no additional cost.

*an additional constraint was imposed: ≥0.0058*Phytates+5.8 (women) or 0.0077*Phytates+7.1 (men). Phytates intakes were (obs, opt): (618,586) in women<50 yo, (683,395) in women 50-64 yo, (654,591) in women ≥65 yo, (821,498) in men <65 yo, (836,459) in men ≥65 yo

**Supplemental Table 4. Percent coverage, in terms of quantity, for indispensable amino acids and total proteins in observed and all modelled diets^1,2^**

| Population | Diet | Histidine | Isoleucine | Leucine | Lysine | Methionine  +Cysteine | Phenylalaline  +tyrosine | Threonine | Tryptophan | Valine | Total proteins |
| --- | --- | --- | --- | --- | --- | --- | --- | --- | --- | --- | --- |
| Women<50y | OBS | 195 | 198 | 156 | 182 | 202 | 243 | 186 | 227 | 226 | 134 |
|  | Model#1 | 126 | 147 | 113 | 117 | 156 | 187 | 133 | 176 | 172 | 105 |
|  | Model#2-NO | 188 | 207 | 162 | 178 | 208 | 262 | 193 | 240 | 242 | 141 |
|  | Model#2-55 | 172 | 194 | 151 | 162 | 197 | 249 | 181 | 227 | 227 | 134 |
|  | Model#2-50 | 158 | 181 | 141 | 146 | 183 | 233 | 166 | 214 | 211 | 127 |
| Women 50-64y | OBS | 186 | 189 | 149 | 176 | 193 | 231 | 179 | 216 | 215 | 127 |
|  | Model#1 | 117 | 133 | 101 | 108 | 138 | 167 | 120 | 156 | 156 | 91 |
|  | Model#2-NO | 207 | 217 | 170 | 194 | 219 | 271 | 206 | 254 | 255 | 145 |
|  | Model#2-60 | 187 | 200 | 156 | 174 | 203 | 252 | 188 | 236 | 233 | 135 |
|  | Model#2-55 | 155 | 176 | 137 | 146 | 180 | 226 | 163 | 208 | 207 | 120 |
|  | Model#2-50 | 149 | 171 | 133 | 139 | 176 | 220 | 158 | 203 | 201 | 118 |
|  | Model#2-45 | 134 | 154 | 119 | 122 | 159 | 198 | 139 | 183 | 179 | 110 |
| Women≥65y | OBS | 150 | 153 | 121 | 141 | 156 | 188 | 144 | 176 | 174 | 104 |
|  | Model#1 | 101 | 119 | 91 | 97 | 122 | 148 | 107 | 143 | 140 | 79 |
|  | Model#2-NO | 164 | 175 | 137 | 156 | 175 | 220 | 165 | 205 | 205 | 117 |
|  | Model#2-60 | 153 | 167 | 130 | 145 | 167 | 211 | 156 | 196 | 194 | 112 |
|  | Model#2-55 | 135 | 153 | 119 | 128 | 154 | 196 | 142 | 179 | 179 | 104 |
|  | Model#2-50 | 124 | 142 | 109 | 117 | 144 | 182 | 130 | 167 | 166 | 99 |
|  | Model#2-45 | 116 | 132 | 102 | 108 | 136 | 171 | 120 | 157 | 154 | 95 |
| Men<65y | OBS | 215 | 213 | 169 | 198 | 219 | 261 | 201 | 246 | 243 | 146 |
|  | Model#1 | 94 | 106 | 81 | 83 | 108 | 135 | 95 | 129 | 123 | 75 |
|  | Model#2-NO | 213 | 225 | 175 | 202 | 228 | 278 | 211 | 259 | 262 | 148 |
|  | Model#2-65 | 202 | 211 | 165 | 188 | 216 | 263 | 198 | 245 | 246 | 141 |
|  | Model#2-60 | 179 | 190 | 149 | 163 | 195 | 240 | 177 | 224 | 221 | 129 |
|  | Model#2-55 | 162 | 171 | 135 | 144 | 173 | 219 | 158 | 206 | 199 | 119 |
|  | Model#2-50 | 139 | 157 | 122 | 124 | 159 | 204 | 142 | 190 | 184 | 109 |
|  | Model#2-45 | 130 | 147 | 114 | 113 | 145 | 190 | 131 | 180 | 170 | 104 |
|  | Model#2-40 | 123 | 136 | 106 | 104 | 139 | 180 | 122 | 170 | 159 | 101 |
| Men≥65y | OBS | 162 | 160 | 128 | 149 | 166 | 198 | 152 | 186 | 184 | 112 |
|  | Model#1 | 80 | 87 | 68 | 69 | 89 | 112 | 79 | 106 | 103 | 61 |
|  | Model#2-NO | 167 | 177 | 137 | 158 | 176 | 219 | 165 | 204 | 207 | 116 |
|  | Model#2-65 | 160 | 170 | 132 | 150 | 170 | 211 | 158 | 196 | 198 | 112 |
|  | Model#2-60 | 141 | 149 | 116 | 128 | 151 | 188 | 139 | 176 | 173 | 101 |
|  | Model#2-55 | 128 | 140 | 109 | 115 | 141 | 178 | 128 | 165 | 162 | 96 |
|  | Model#2-50 | 117 | 128 | 100 | 104 | 128 | 165 | 116 | 153 | 149 | 90 |
|  | Model#2-45 | 109 | 119 | 93 | 95 | 121 | 156 | 108 | 144 | 139 | 86 |
|  | Model#2-40 | 101 | 112 | 88 | 87 | 117 | 148 | 102 | 138 | 130 | 87 |

^1^ OBS=Observed diet ; Models#1= models focused on determining the theoretical minimum level of total dietary proteins that is compatible with the fulfilment of all nutrient-based recommendations (without applying a minimum total protein quantities) without increasing total energy content and at no additional cost; Model#2-NO= models focused on determining the minimum departure to observed diet that is compatible with the fulfilment of all nutrient-based recommendations (without applying a minimum total protein quantity) without increasing total energy content, at no additional cost and with no constraint on the animal protein contribution; Model#2-XX=models aimed at determining the minimum departure to observed diet that is compatible with the fulfilment of all nutrient-based recommendations (without applying a minimum total protein amount) without increasing total energy content, at no additional cost and with a constraint applying a contribution in animal proteins that is XX% of total proteins

^2^ red cells indicate a value below 100 meaning that the indispensable amino acid or protein requirement is not achieved.

**Supplemental Figure 1. Value of the objective function (i.e. total departure from observed and modelled diets) expressed in grams/d (A) and total number of foods (B) in diets with the set of Model Set#2^1^ without (MOD2-NO) and with a progressively decreasing constraint in 5% steps to the animal protein contribution in total proteins, until no solution could be found, for each of the five subpopulations**


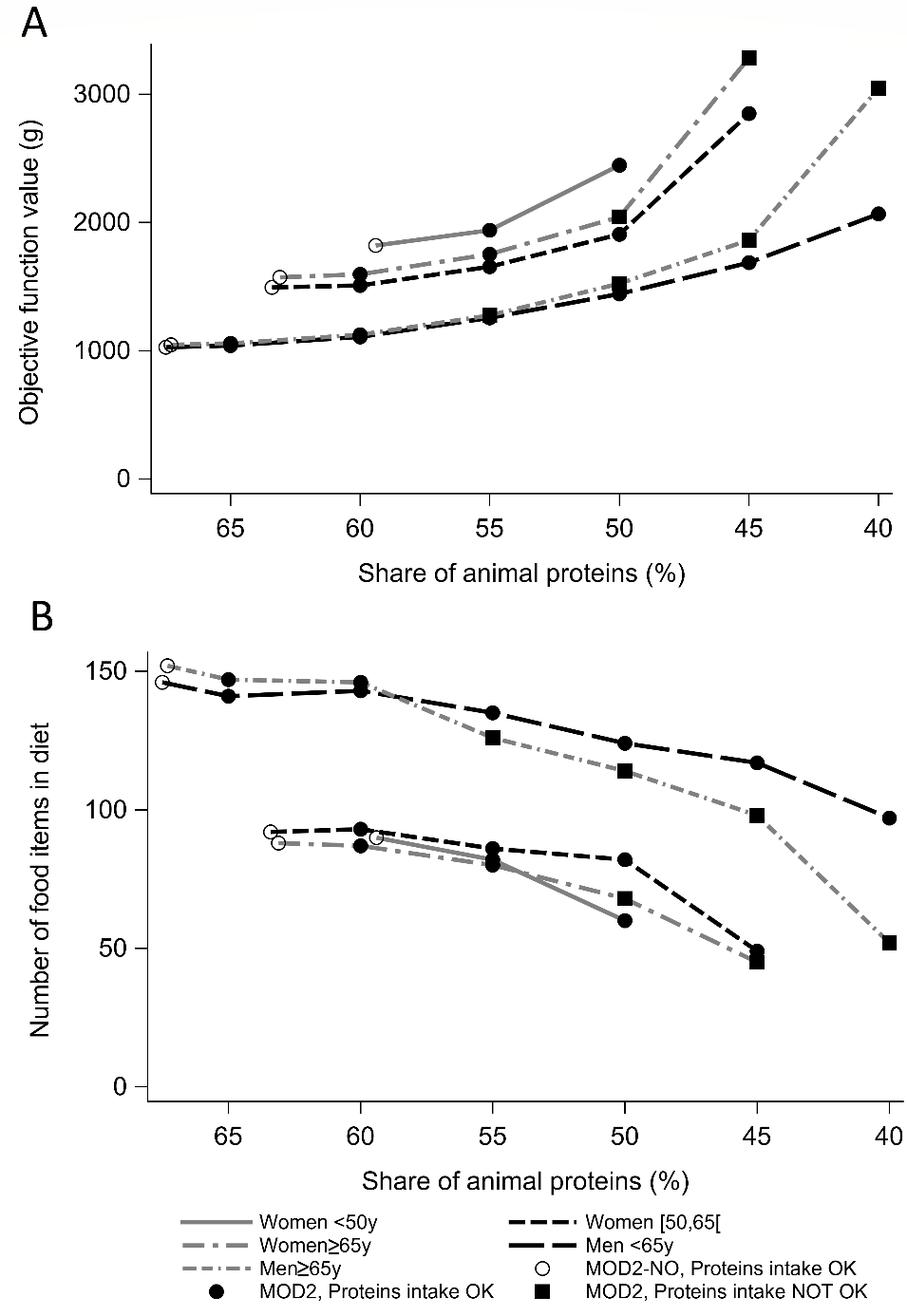


**Footnote:** ^1^ Models imposing the fulfilment of all nutrient-based recommendations except for the one for proteins, while minimizing the departure from the observed diet

Squares indicate that the modelled diet does not contain the recommended level of proteins.
